# The Association Between Obstructive Sleep Apnea and Arrhythmias

**DOI:** 10.7759/cureus.4429

**Published:** 2019-04-10

**Authors:** Avani R Patel, Amar R Patel, Shivank Singh, Shantanu Singh, Imran Khawaja

**Affiliations:** 1 Internal Medicine, Northern California Kaiser Permanente, Fremont, USA; 2 Internal Medicine, Maoming People's Hospital, Maoming, CHN; 3 Pulmonary Medicine, Marshall University School of Medicine, Huntington, USA

**Keywords:** obstructive sleep apnea, arrhythmias, atrial fibrillation, continuous positive airway pressure therapy

## Abstract

Obstructive sleep apnea (OSA) is caused by intermittent episodes of partial or complete closure of the upper airway, leading to apneic episodes while the patient is asleep. Atrial fibrillation (AF) leads to more than 750,000 hospitalizations per year and accounts for an estimated 130,000 deaths each year. The death rate from AF as the primary or a contributing cause of death has been rising for more than two decades. The material reviewed in this paper focuses on the association between OSA and arrhythmias. It goes into the details of the epidemiology, pathophysiology, and types of arrhythmias and the therapies seen in association with OSA.

## Introduction and background

Sleep-disordered breathing (SDB) is a cumulative term for sleep-related breathing disorders and abnormalities of respiration during sleep. SDB consists of obstructive sleep apnea (OSA), central sleep apnea syndrome, sleep-related hypoventilation disorder, sleep-related hypoxemia, primary snoring, and nocturnal groaning. OSA is caused by intermittent episodes of partial or complete closure of the upper airway, leading to apneic episodes while the patient is asleep [1]. OSA is diagnosed by a combination of positive patient history and positive findings on polysomnography. An apnea-hypopnea index (AHI) of greater than five is diagnostic of OSA.

## Review

Epidemiology

The incidence and prevalence of OSA vary based on age in the general population. The prevalence is higher in the middle-aged and 65-years and above populations [2]. Based on an AHI of more than five, the prevalence ranges from 9%-38% and is higher in men than in women [2]. At an AHI of more than 15, the prevalence in the general adult population ranged from 6%-17% and was 49% in the 65-years and above group [2].

Atrial fibrillation (AF) has a prevalence of 9% in the 65-year-old and above population as compared to the 2% prevalence in the younger than 65-year-old population [3]. An estimated 2.7-6.1 million people in the United States have AF [3]. AF leads to more than 750,000 hospitalizations per year and accounts for an estimated 130,000 deaths each year [4-5]. Medical costs for AF patients are about $8,705 higher than for people without AF [3,6]. The death rate from AF as the primary or a contributing cause of death has been rising for more than two decades [4-5].

Pathophysiology

Over the years, attempts have been made to determine how arrhythmias develop in OSA patients. In OSA, the frequent collapse of the airway causes oxyhemoglobin desaturation, which leads to persistent inspiratory efforts made against a collapsed airway, often resulting in the patient’s arousal from sleep [1].

One hypothesis is that OSA causes reduced blood oxygen and increased carbon dioxide levels due to problems with the baroreflex and chemoreflex activities, leading to the activation of the sympathetic nervous system, causing electrical remodeling of the heart [7-10]. This remodeling can lead to arrhythmia development [1,7-10].

Another hypothesis is that co-existing hypertension in OSA patients may be responsible for the development of AF. Past studies have shown a strong association between OSA and hypertension, as well as hypertension and AF. Hypertension has been shown to cause atrial remodeling. Similarly, in OSA patients with hypertension, it is hypothesized that persistent inspiratory efforts made against a collapsed airway cause dramatic shifts in intracardiac pressures, leading to the activation of atrial ion channels, thus creating an acute change in cardiac chamber dimensions [11-12]. This change in size can lead to AF development [11-12].

The third hypothesis is the OSA effect of applying negative intrathoracic pressure on both atrial and ventricular free walls, which will lead to cardiac stretching, thus activating cardiac ion channels, causing a change in cardiac chamber size, which can lead to arrhythmias [13].

The fourth hypothesis is related to OSA causing hypoxemia that stimulates the vagal nerve, leading to cardiac vagal reflex activation. In approximately 10% of OSA patients, the resulting hypoxemia activates the cardiac vagal reflex, leading to the development of bradyarrhythmias even in the absence of cardiac conduction disease [14].

Types of arrhythmias

Different types of arrhythmias may be found in patients of OSA. Based on past research studies (see Table 1), there are several observed associations between OSA and arrhythmias.

**Table 1:** Summary of Incidence and Prevalence of OSA and Arrhythmias OSA: obstructive sleep apnea; AF: atrial fibrillation, HF: heart failure; SAS: sleep apnea syndrome; SCD: sudden cardiac death; LVEF: left ventricular ejection fraction; AHI: apnea-hypopnea index

Name and Year of Study	Type of Study	Number of Participants	Outcome Considered	Arrhythmia Relationship with OSA
Atrial fibrillation				
Mehra et al. 2006 [15]	Longitudinal cohort	566	Finding out the prevalence of nocturnal cardiac arrhythmias in sleep disordered breathing patients.	Study contained 228 patients with OSA and 338 patients without OSA. AF had a prevalence of 4.8% in the study population. It was determined that severe OSA will have a two-to-four fold higher chance of developing complex arrhythmias.
Gami et al. 2004 [16]	Prospective, Cross-sectional	524	Finding out the prevalence of OSA in AF patients.	Study contained 151 patients with AF and 312 patients with another cardiovascular disease (CVD). The adjusted odds ratio was 2.19. The prevalence of OSA was higher in patients with AF than in the opposing group.
Porthan et al. 2004 [17]	Case-control	115	Finding out the prevalence of arrhythmias in OSA patients.	OSA was common in AF patients. The study could not demonstrate that OSA was more common in AF patients than in the corresponding controls.
Javaheri et al. 1998 [18]	Prospective	81	Finding out the prevalence of arrhythmias in OSA patients.	AF had a prevalence of 32% in the study population. All patients were male, ambulatory, with stable HF, and had LVEF below 45%.
Flemons et al. 1993 [19]	Prospective	263	Finding out the prevalence of arrhythmias in OSA patients.	Patients having sleep apneas were found to have a low prevalence of arrhythmias. There was a 1.3% prevalence of complex ventricular ectopy, a 2.6% prevalence of frequent ventricular premature beats, 1.3% prevalence of second-degree atrioventricular block, and a 5.2% prevalence of sinus arrest.
Mooe et al. 1996 [20]	Prospective cohort	121	Finding out the prevalence of arrhythmias in OSA patients.	AF had a 32% prevalence in patients with AHI more than five. Furthermore, AF was found with an 18% prevalence in patients with AHI less than five.
Sick sinus syndrome				
Simantirakis et al. 2004 [21]	Longitudinal	23	Finding out the prevalence of arrhythmias in OSA patients.	Rhythm disturbances had a prevalence of 48% in the population. They consisted of frequent episodes of bradycardia and long pauses, which were observed in patients who had moderate to severe OSA.
Garrigue et al. 2007 [22]	Observational	98	Finding out the prevalence and consequence of sleep apnea syndrome in pacemaker patients.	There was a 59% prevalence of undiagnosed OSA in pacemaker patients.
Grimm et al. 1995 [23]	Observational	12	Assessing the sinus and AV node abnormalities in OSA.	The study was conducted with patients who had ventricular asystole and OSA. It was concluded that there was no significant association of sinus node and AV node abnormalities with OSA.
Steiner et al. 2008 [24]	Observational	12	Understanding the relation between sleep apnea and sinus abnormalities.	All patients had heart failure. There was no correlation observed between patients having mild sleep apnea and sinus abnormalities.
Velasco et al. 2014 [25]	Observational	190	Defining the association between severe OSA and symptomatic bradyarrhythmias.	The Berlin questionnaire was used during the study. It was concluded that patients at high risk for OSA did not have an increased prevalence of bradyarrhythmias.
Atrioventricular block				
Becker et al. 1995 [26]	Observational	239	Finding out the prevalence of arrhythmias in OSA patients.	In about 30% of patients with sleep apnea, there was sinus arrest and AV block.
Sudden cardiac death				
Gami et al. 2013 [27]	Prospective, longitudinal	10,701	Understanding the association between OSA and SCD.	It was determined that compared to the general population, SCD is prevalent in OSA patients. During the average follow-up of 5.3 years, 142 patients were either resuscitated or had fatal SCD with an annual rate of 0.27%. This is more than that in the general population where the annual rate is approximately less than 0.1%.
Gami et al. 2005 [28]	Retrospective	112	Understanding the association between OSA and SCD.	112 death certificates were reviewed. All had died from SCD. Of the SCD patients, there was a prevalence of 46%.

Atrial fibrillation is a commonly seen arrhythmia in OSA patients [1]. AF occurs when disordered atrial electric activity causes an abnormal electrical rhythm that replaces the normal sinus mechanism [29]. It can be caused by hypertension, myocardial infarction, hyperthyroidism, caffeine use, and abnormal heart valves and is seen in sleep apnea patients. AF often goes undiagnosed for a long time because 10%-40% of AF patients are asymptomatic [30]. Symptomatic patients will present with palpitations, shortness of breath, exercise intolerance, chest pain, or malaise [29]. AF is responsible for an estimated 130,000 deaths per year and for the worsening morbidity in other diseases like stroke and heart failure [4-5,30]. AF is found in 2% of the general population, with an increasing prevalence to 9% in the above 65 years population [3,30].

Sick sinus syndrome (SSS) refers to a collection of disorders marked by the heart's inability to perform its pacemaking function [31]. SSS mostly affects older adults and consists of bradyarrhythmias with or without accompanying tachyarrhythmias [31]. At least 50% of SSS patients develop alternating bradycardia and tachycardia, also known as Tachy-Brady syndrome [31]. SSS results from degenerative fibrosis, ion channel dysfunction, and the remodeling of the sinoatrial node [31]. Signs and symptoms are often subtle early on and become more obvious as the disease progresses [31]. They are commonly related to end-organ hypoperfusion, like syncope secondary to cerebral hypoperfusion [31].

Atrioventricular block (AV block) is an arrhythmia that is caused by a delay or disturbance in the transmission of an electrical impulse from the atria to the ventricles [32]. This can be due to an anatomical or functional impairment in the heart’s conduction system [32]. In general, there are three degrees of AV nodal blocks: first-degree, second-degree (Mobitz type 1 or 2), and third-degree [32]. The causes of AV blocks are myocardial infarction, post-cardiac surgery, electrolyte imbalances, idiopathic fibrosis, and medications that slow atrioventricular conduction [32]. Patients may be asymptomatic or they may present with palpitations, syncope, and dizziness.

Sudden cardiac death is defined as natural death due to cardiac causes, which will present as an abrupt loss of consciousness within the first hour of symptoms [33]. The mechanisms can be ventricular fibrillation, ventricular tachycardia, and flutter with subsequent ventricular fibrillation, torsades de pointes, and, lastly, bradyarrhythmias and asystolic arrest [33].

Treatment

There are no conclusive epidemiologic or longitudinal intervention studies that relate specifically to the prevalence, severity, and consequences of cardiac arrhythmias and the effects of OSA treatment [1]. Despite this, there have been many observations made from previous studies regarding the effectiveness of continuous positive airway pressure (CPAP) therapy for OSA patients and their therapeutic effect on arrhythmia incidence in the same patients. Based on the studies (see Table 2), CPAP therapy had an effect on reducing the incidence and prevalence of cardiac arrhythmias in OSA patients.

**Table 2:** Therapeutic Effect of CPAP Treatment on Patients with Both Arrhythmias and OSA AHI: apnea-hypopnea index; CPAP: continuous positive airway pressure; OSA: obstructive sleep apnea; VPB: ventricular premature beats

Author Name and Year of Study	Type of Study	Study Population	CPAP Therapy Relationship with Reducing Arrhythmia Occurrence
Kufoy et al. 2012 [34]	Retrospective, Cross-sectional	39 patients	Heart rate variability went from a basal value $0.0673\pm 0.011​​​​$​​​ to a post-CPAP therapy value $0.0935\pm 0.022$. It was determined that after only one night of CPAP treatment, patients with significant cases of OSA experienced a substantial resolution of heart rate variability.
Becker et al. 1995 [26]	Observational	239 patients total	It was revealed that 7% of 239 (17 patients) with OSA had significant bradyarrhythmias and of these 17 patients, only one continued to experience bradyarrhythmias after CPAP therapy. Overall, a 95% reduction in bradyarrhythmias occurrence after CPAP therapy.
Simantirakis et al. 2004 [21]	Longitudinal	23 patients	Before CPAP therapy was initiated, there was a 47% prevalence of cardiac rhythm disturbances. Six months after treatment, only two patients had cardiac rhythm disturbances. Overall, there was a 91% reduction in cardiac rhythm disturbances after CPAP therapy.
Ryan et al. 2005 [35]	Randomized Control Trial	18 patients	The results showed that the treatment of OSA in those patients reduced the frequency of VPB by 58% during sleep.
Kurlykina et al. 2009 [36]	Cross-sectional	19 patients	CPAP therapy caused AHI to decrease from 60.7 episodes per hour to only 5.5 episodes per hour - a 91% reduction in AHI post-CPAP therapy.
Kanagala et al. 2003 [37]	Observational	43 patients	An increased rate of recurrence (82%) of AF after successful cardioversion in OSA patients inadequately treated with CPAP therapy, as compared with non-OSA patients and OSA patients treated adequately with CPAP.
Harbison et al. 2000 [38]	Prospective, Cross-sectional	45 patients	The treatment results showed a complete resolution of previously observed rhythm disturbances in seven out of eight patients. A 88% reduction in rhythm occurrence in post-CPAP therapy patients.
Marin et al. 2005 [39]	Prospective, Cohort	1,651 total patients	It was determined that patients with untreated severe disease had a higher incidence of fatal cardiovascular events (1.06 per 100 person-years) and non-fatal cardiovascular events (2.13 per 100 person-years) than did untreated patient with mild-moderate disease (0.55, p=0.02 and 0.89, $p&lt;0.0001$​​​​​​​), simple snorers (0.34, p=0.0006 and 0.58, $p&lt;0.0001$​​​​​​​), CPAP-treated patients (0.35, p=0.0008, and 0.64, $p&lt;0.0001$​​​​​​​), and healthy participants (0.3, p=0.0012, and 0.45, $p&lt;0.0001$​​​​​​​).

## Conclusions

The material reviewed in this paper focuses on the association between OSA and arrhythmias. It goes into the details regarding the epidemiology, pathophysiology, and types of arrhythmias seen in association with OSA. It also addresses observations regarding CPAP therapy in reducing arrhythmias. Despite these key points being addressed, larger and more prospective studies are needed to understand the true benefits of CPAP therapy. This is a review article for busy, practicing physicians to have a cumulative view of our current situation regarding the need for CPAP therapy in patients of both OSA and arrhythmias.
